# RETRACTION: A Meta‐Analysis on the Impact of Spinal Cord Stimulation on Post‐Operative Wound Healing in Patients With Multiple Sclerosis

**DOI:** 10.1111/iwj.70581

**Published:** 2025-04-18

## Abstract

**RETRACTION:** CaiY. , ZhengX. , and GanF. , “A Meta‐Analysis on the Impact of Spinal Cord Stimulation on Post‐Operative Wound Healing in Patients With Multiple Sclerosis,” International Wound Journal 21, no. 2 (2024): e14719, 10.1111/iwj.14719.PMC1082756542053047

The above article, published online on 30 January 2024, in Wiley Online Library (http://onlinelibrary.wiley.com/), has been retracted by agreement between the journal Editor in Chief, Professor Keith Harding; and John Wiley & Sons Ltd. Following an investigation by the publisher, all parties have concluded that this article was accepted solely on the basis of a compromised peer review process. The editors have therefore decided to retract the article. The authors disagree with the retraction.

**RETRACTION:** Y. Cai , X. Zheng , and F. Gan , “A Meta‐Analysis on the Impact of Spinal Cord Stimulation on Post‐Operative Wound Healing in Patients With Multiple Sclerosis,” International Wound Journal 21, no. 2 (2024): e14719, 10.1111/iwj.14719.PMC1082756542053047

The above article, published online on 30 January 2024, in Wiley Online Library (http://onlinelibrary.wiley.com/), has been retracted by agreement between the journal Editor in Chief, Professor Keith Harding; and John Wiley & Sons Ltd. Following an investigation by the publisher, all parties have concluded that this article was accepted solely on the basis of a compromised peer review process. The editors have therefore decided to retract the article. The authors disagree with the retraction.

